# STAT3-NAV2 axis as a new therapeutic target for rheumatoid arthritis via activating SSH1L/Cofilin-1 signaling pathway

**DOI:** 10.1038/s41392-022-01050-7

**Published:** 2022-07-08

**Authors:** Ran Wang, Jianghong Cai, Keyuan Chen, Menglin Zhu, Zhaoyi Li, Hua Liu, Tiantian Liu, Jianchun Mao, Qian Ding, Yi Zhun Zhu

**Affiliations:** 1 State Key Laboratory of Quality Research in Chinese Medicine, School of Pharmacy, Macau University of Science and Technology, Avenida Wai Long, Taipa, Macau China; 2 School of Pharmacy, Minzu University of China, Beijing, 100081 China; 3 Department of Pharmacy, Aerospace Center Hospital, Beijing, 100049 China; 4 Department of Rheumatology, Longhua Hospital of Shanghai University of Traditional Chinese Medicine, Shanghai, 200032 China; 5 Shanghai Key Laboratory of Bioactive Small Molecules, Department of Pharmacology, School of Pharmacy, Fudan University, Shanghai, 201203 China

**Keywords:** Inflammation, Rheumatic diseases

**Dear Editor**,

Rheumatoid arthritis (RA), which is the most common inflammatory arthritis disease, as well as a kind of most prevalent autoimmune disease, affects 1–3% population all over the world.^1^ Currently available treatments can reduce the disability rate of patients with RA to some extent, but they cannot completely block the inflammatory joint destruction and relieve the pain that comes with it. Our previous study found that Neuron Navigator 2 (NAV2) increased significantly in RA and was regulated by transcription factor E2F1.^2^ However, it is still unclear why NAV2 is elevated at the genetic level in RA fibroblast-like synoviocytes (FLS). Additionally, RA’s exact pathological mechanisms and therapeutic targets are also not fully understood. Hence, an in-depth study of the pathogenesis of RA is urgently needed.

In this study, super-enhancer (SE) identification in RA FLS was performed through ChIP-seq against the H3K27ac modification. SE refers to extra-long *cis*-acting elements of 8–20 kb in length that have transcriptional enhancement activity, which can assemble key transcription factors and cofactors in high density and activate the expression of identity determining genes in stem cells. Thus, SE plays an important role in the modulation of cells fate and can be recognized as a valuable biomarker and therapeutic target to identify and intervene with disease-related genes.^3^ The results displayed that ﻿significantly increased ChIP-seq signal of H3K27ac in human RA FLS (CHIPMD) when compared to human osteoarthritis (OA) FLS (CHIPCK) (Supplementary Fig. S1a, b). The enrichment level of H3K27ac in the coding gene region showed that the overall level of H3K27ac increased significantly in RA FLS (Supplementary Fig. S1c). Moreover, we also found that in human RA FLS, the signal strength of SE was significantly stronger than typical enhancers (Supplementary Fig. S1d). Compared with 642 SE-associated genes identified in OA FLS, 1368 SE-associated genes were identified in RA FLS which indicated that more genes were driven to express in RA to promote disease progression (Supplementary Fig. S1e, f). GO analysis and KEGG pathway analysis were used to investigate the functional enrichment of the identified SE-related targets and pathways (Supplementary Fig. S2a, b). The SEs were associated with genes enriched in axon guidance, cell adhesion, and cell motility. Additionally, regulation of the actin cytoskeleton pathway could be stimulated in RA. These results indicated that the SE-associated genes and signaling pathways in RA FLS were hyperactive and may promote the progression of RA. Interestingly, the SE signal of NAV2 showed a significant increase in primary human RA FLS (Supplementary Fig. S3). Then the results of the expression of Vimentin showed that the cells derived from synovium tissues were primary FLS and the expression of NAV2 was markedly upregulated by using an immunofluorescence double staining experiment (Fig. 1a). Also, the obvious increase of NAV2 was confirmed in the primary synovial cells from RA synovium samples through Western blot analysis (Fig. 1b). Next, we found that the expression of NAV2 was significantly elevated in AIA rat synovial tissues. The clinical symptoms were evaluated every 5 days from Day 10 to Day 30. Images of rats’ hind paws displayed remark inflammatory exacerbation and soft tissue swelling as time went on. Micro-CT deeply showed the typical symptoms of bone destruction, swollen joints, synovial membrane hyperplasia with severe infiltration of inflammatory cells, as well as pannus formation in joint tissues (Supplementary Fig. S4a). Arthritis scores and hind paw volumes also showed more severity and higher incidence of arthritis in the model group when compared to the control group (Supplementary Fig. S4b, c). These results indicated that the AIA rats model was successfully established in this study. Interestingly, the NAV2 expression was also remarkedly increased in the inflamed joints on the protein level (Fig. 1c). In previously oncology-related studies, NAV2 expression has been found to be increased in cancers such as colon cancer and uterine sarcoma. Moreover, the overexpression of NAV2 is also associated with the poor prognosis of colorectal cancer (CRC) and promotes CRC invasion through the slingshot-1L (SSH1L)/Cofilin-1 signaling pathway.^4^ So we speculated that NAV2 could regulate this pathway in RA. We first showed that the SSH1L/Cofilin-1 signaling pathway was activated obviously in inflamed joints of AIA rats (Fig. 1c).

**Fig. 1:**
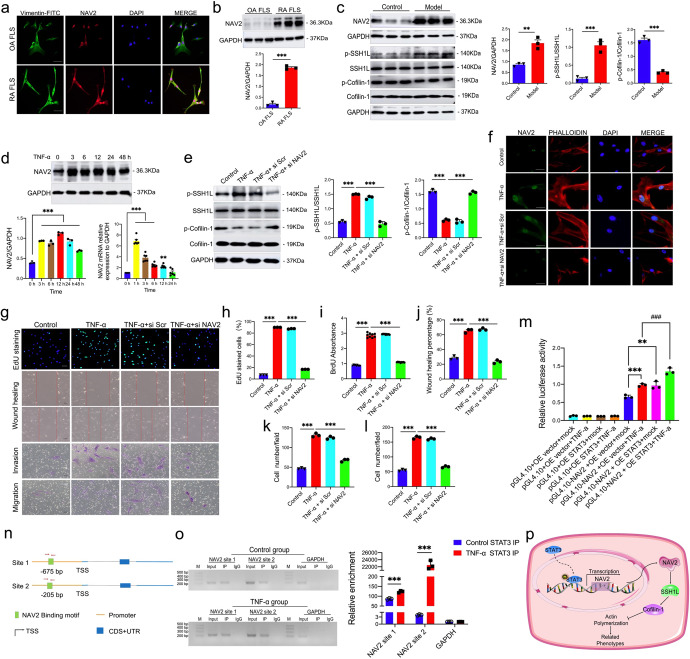
Transcription factor STAT3 regulates NAV2 expression to promote FLS proliferation, migration, and invasion in RA. **a** Immunofluorescence double staining analysis for the expression of NAV2 and Vimentin. DAPI for stain nuclei (blue). Vimentin and NAV2 were stained with green and red, respectively. **b** Expression of NAV2 level in FLS were examined. **c** Western blotting for NAV2, p-SSH1L, SSH1L, p-Cofilin-1, and Cofilin-1 in rats synovium tissue. Data were presented as means ± SEM from at least three independent experiments. *n* = 6–8 rats per group. Scale bars indicate 50 μm for immunohistochemistry. **P < 0.01, ***P < 0.001 vs Control group. Human RA FLS were treated with TNF-α (20 ng/ml) for 0, 1, 3, 6, 12, 24, and 48 h, the analysis of the inflammatory mediators was described in Materials and methods, respectively. **d** The expression level of NAV2 in RA FLS, at the different times following TNF-α treatment, was determined by Western blot and qRT-PCR. Data were presented as mean ± SEM of more than three independent experiments. **P < 0.01, ***P < 0.001 vs unstimulated cells. FLS were transfected with si Scr or si NAV2 prior to treatment with TNF-α (20 ng/ml), the level of protein expression was determined by Western blotting after 30 min or 12 h stimulation. **e** Silencing NAV2 decreased phosphorylation of SSH1L and increased phosphorylation of Cofilin-1 in contrast to control cells, GAPDH serves as the internal control. **f** Cells were subjected to immunofluorescence staining for NAV2 and PHALLOIDIN. DAPI for stain nuclei (blue). NAV2 and PHALLOIDIN were stained by green and red, respectively. Scale bars, 50 μm. **g**, **h** EdU incorporation with the quantification of percent EdU^+^ cells in NAV2 knockdown (siRNA) and control (si Scr) FLS before and after TNF-α treatment. **i** BrdU absorbance analysis. **g**, **j** Scratch wound migration assay showed that FLS had an augmented ability to migrate when compared to the control cells. **g**, **k**, **l** Transwell results showed that silencing NAV2 expression could obviously impede the invasion and motility of the cells. All images shown are representative ones from at least three replicates, results shown as mean ± SEM, ***P < 0.001 (*n* ≥ 3). **m** Effect of STAT3 on NAV2 gene promoter activity. A luciferase assay was carried out to analyze NAV2-dependent transcriptional activity by using 293 T cells. Data were presented as mean ± S.D, *n* = 3, ***P < 0.001 vs OE vector+mock group, ^###^*P* < 0.001 vs OE vector+TNF-α group. **n**, **o** Schematic diagram of two pairs of primers designed for ChIP spanning the NAV2 promoter, STAT3 bound to the NAV2 promoter indicated by ChIP in RA FLS. IgG from rabbits served as a control. Data were presented as means ± SEM of three independent experiments. **P < 0.01, ***P < 0.001. **p** Working model for STAT3-NAV2 axis accelerates inflammatory response and related phenotypes. Unstimulated RA FLS has a lower level of NAV2 protein. NAV2 protein level increased after TNF-α-induced, leading to inflammatory protein response and inflammation-associated phenotypes. NAV2 was upregulated by transcription factor STAT3 and then activated the SSH1L/Cofilin-1 signaling pathway, subsequently promoting the progress of RA

Human RA FLS were treated with TNF-α (20 ng/ml) for 0, 1, 3, 6, 12, 24, and 48 h. As illustrated in Supplementary Fig. S5a–e, protein expression levels of IL-6, iNOS, MMP-3, and MMP-9 were significantly increased time-dependently. Among the results, the protein levels of IL-6, iNOS, and MMP-9 showed a persistent significant upregulation from 0 to 12 h, whereas MMP-3 protein expression reached a peak at 24 h during TNF-α-induced cellular inflammation. Similarly, the results also showed the elevated mRNA levels of iNOS and IL-8 (Supplementary Fig. S5f, g). Collectively, the results indicated that TNF-α-stimulated FLS exhibited an activated inflammatory response state, similar to the increased pro-inflammatory mediators to active the RA external environment. Interestingly, the NAV2 mRNA level reached a peak at 1 h and then declined. Consistent with this, the expression of NAV2 protein showed a significant increase during TNF-α-induced cellular inflammation from 0 to 24 h but slightly decreased at 48 h (Fig. 1d), the peak of NAV2 might indicate NAV2 played a crucial role in the initial phase of RA progression.

NAV2 is a protein-coding gene that plays an important role in cellular growth, migration, and invasion. As illustrated in Supplementary Fig. S6a–e, after knockdown of NAV2 expression by transfection with siRNA, we could find a decrease in NAV2, accompanied by the expression of COX-2, IL-6, and MMP-9 decreased obviously. Besides, silenced NAV2 significantly decreased the mRNA level of iNOS (Supplementary Fig. S6f). More importantly, the results of immunofluorescence double staining also showed that TNF-α induced the co-expression of iNOS and NAV2 was eliminated after NAV2 was knocked down (Supplementary Fig. S7). Taken together, these findings strongly suggested that silencing NAV2 was sufficient to partly antagonize the inflammatory response in RA.

In vitro experiment, the results showed that the abundance of phosphorylated SSH1L (p-SSH1L) was significantly increased upon TNF-α treatment and phosphorylated Cofilin-1 (p-Cofilin-1) decreased dramatically (Supplementary Fig. S8a), indicating that TNF-α probably activates SSH1L/Cofilin-1 signaling pathway through phosphorylation activation. Additionally, we also found that knockdown of the NAV2 gene could dramatically decrease p-SSH1L but increase p-Cofilin-1 in contrast to control cells (Fig. 1e). Previous results showed that the migration and invasion of FLS were related to NAV2 expression in RA.^2^ Here, our results showed a decrease in F-actin polymerization and stress fiber disassembly in cells with silenced NAV2 gene (Fig. 1f). In addition, after downregulated NAV2 expression, the NAV2 knockdown (NAV2-KD) cells showed decreased TNF-α-induced EdU incorporation and BrdU absorbance (Fig. 1g–i). Moreover, the Scratch wound migration assay showed that FLS had an augmented ability to migrate when compared to the control cells. Transwell assay also showed that silencing NAV2 expression could obviously impede the invasion and motility of the cells (Fig. 1g, j–l). These experiments revealed that ﻿NAV2 plays a crucial role in promoting invasion and metastasis of RA FLS by modulating F-actin polymerization through the SSH1L/Cofilin-1 signaling pathway.

The underlying mechanisms by which NAV2 accelerates inflammatory response and associated phenotypes in RA were investigated in the following study. First, we showed that TNF-α time-dependently increased phosphorylated STAT3 (p-STAT3) significantly in human RA FLS (Supplementary Fig. S8b). Then it was interesting to find that overexpressed STAT3 in FLS resulted in upregulating NAV2 expression, whereas knockdown of STAT3 had the opposite effect (Supplementary Fig. S9a, b). To further clarify that STAT3 regulates the expression of NAV2 and then promotes the inflammation reaction in RA we performed Luciferase reporter assay and ChIP assay. Of note, elevated STAT3 expression could dramatically enhance the transcription of NAV2 (Fig. 1m), and ChIP results showed that STAT3 could enrich the promoter region of NAV2 from −2000 to +500 (Fig. 1n, o). More importantly, according to the binding mode, it can be clearly seen that NAV2 and STAT3 proteins match well (the binding energy is −65.05 kcal/mol, Table S1). These hydrogen bonds can effectively bind the two proteins tightly to form a stable complex (Supplementary Fig. S10). RMSD measures the average distance between atoms to reveal structural changes during the simulation over time. Lower deviations of the RMSD value mean better stability. The RMSD value of the STAT3-NAV2 complex was significantly reduced and maintained at about 0.4 nm, indicating excellent interaction efficiency and stability. Collectively, computational docking and molecular dynamics simulation both suggest that transcription factor STAT3 can tightly bind to NAV2 (Supplementary Fig. S11).

Collectively, we present compelling evidence that NAV2 is a novel regulator of primary human RA FLS in proliferation, migration, and invasion. Our research firstly demonstrated that the inhibition of NAV2 expression in the RA FLS could reverse the inflammation-related phenotypes and prevent RA progression through regulating the SSH1L/Cofilin-1 signaling pathway, supporting a potential role for NAV2 and its regulator STAT3 in a variety of autoimmune diseases (Fig. 1p). As chronic pain is a major feature in RA, we also conjecture that targeting this axis may interfere with the sensitization of joint-innervating neurons that actuate pains in RA.^5^ Therefore, the STAT3-NAV2 axis as a potential molecular target provides an attractive novel factor for interventions to preserve inflammatory diseases such as RA.

## Supplementary information

STAT3-NAV2 axis as a new therapeutic target for rheumatoid arthritis via activating SSH1L/Cofilin-1 signaling pathway

## Data Availability

The data sets generated during and/or analyzed during the current study are available from the corresponding author upon reasonable request.
