# Supplementary material for: STAT3-NAV2 axis as a new therapeutic target for rheumatoid arthritis via activating SSH1L/Cofilin-1 signaling pathway
Source: Signal Transduct Target Ther. 2022 Jul 8;7:209. doi: 10.1038/s41392-022-01050-7 (PMC9262938; doi:10.1038/s41392-022-01050-7)
Supplement: Supplementary file 1 — STAT3-NAV2 axis as a new therapeutic target for rheumatoid arthritis via activating SSH1L/Cofilin-1 signaling pathway [file 41392_2022_1050_MOESM1_ESM.docx]

Supplementary Materials for

**STAT3-NAV2 axis as a new therapeutic target for rheumatoid arthritis via activating SSH1L/Cofilin-1 signaling pathway**

Ran Wang, Jianghong Cai, Keyuan Chen, Menglin Zhu, Zhaoyi Li, Hua Liu, Tiantian Liu, Jianchun Mao, Qian Ding, Yi Zhun Zhu

Correspondence to: Yi Zhun Zhu (yzzhu@must.edu.mo)

**This PDF file includes:**

Materials and Methods

Figures. S1 to S11

Table. S1 to S2

**Materials and Methods**

**Reagents**

Recombination human TNF-α was from Peprotech (NJ, USA). Complete Freund’s adjuvant (CFA, heat-inactivated Mycobacterium tuberculosis) was obtained from Chondrex (WA, USA). Antibodies were purchased from the following commercial sources: Signal Transducer and Activator of Transcription 3 (STAT3), Inducible Nitric Oxide Synthase (iNOS), Cyclooxygenase-2 (COX-2), Interleukin-6 (IL-6), MMP-3, MMP-9 and glyceraldehyde-3-phosphate dehydrogenase (GAPDH) were purchased from Santa Cruz Biotechnology, Inc. (CA, USA); SSH1L, p-SSH1L, Cofilin-1, p-Cofilin-1, p-STAT3 were obtained from Cell Signaling Biotechnology (MA, USA); Neuron Navigator 2 (NAV2) was obtained from Abnova (Taipei, Taiwan). EpiTM chromatin immunoprecipitation kit (Epibiotek, Guangzhou, China); EpiTM DNA Clean Beads (Epibiotek, Guangzhou, China); QIAseq Ultralow Input Library Kit (Qiagen, Germany).

**Animal model of adjuvant-induced arthritis (AIA) rats**

30 SPF grade Male Sprague-Dawley (SD) rats (180-200 g) were from Beijing Vital River Laboratory Animal Technology Co. Ltd (Beijing, China), randomized into 2 groups of 15 animals each. The rats were given 7 days to adapt to the laboratory environment before the experiments began. Adjuvant arthritis was induced according to the method described previously.^1^ All animals were under a controlled temperature (23 ± 1°C) and humidity (50% ± 5%) with a 12 h light/dark cycle and free access to the standard food and water. The body weight, arthritis scores, and hind paw volume were measured by two independent observers on the 0, 10, 15, 20, 25, 30th day after the injection by a plethysmometer. Then, the rats were sacrificed by cervical dislocation. All experimental processes were carried out within the approved guidelines of the Ethics Review Board for Animal Research, Macau University of Science and Technology.

**Cell culture and treatment**

Human embryonic kidney (HEK) 293T cells were obtained from the American Type Culture Collection were maintained in Dulbecco’s Modification of Eagle’s Medium (DMEM) supplemented with 10% fetal bovine serum (FBS, Gibco, MA, USA) and 1% penicillin-streptomycin (Gibco, MA, USA) at 37℃ in a 95% air/ 5% CO_2_ incubator. Fibroblast-like synoviocytes (FLS) are the main effector cells of RA joint synovitis and joint destruction. They can be activated by the pro-inflammatory cytokines IL-1β or TNF-α and produce a large number of pro-inflammatory mediators. Among them, iNOS, IL-6 and COX-2 are conventional pro-inflammatory mediators, who play a significant role in the onset and development of RA.^2^ Primary human FLS were isolated from the synovial tissues by using the collagenase digestion method as previously described ^1,3^ and cultured in DMEM supplemented with 15% FBS and 1% penicillin-streptomycin at 37℃ in a 95% air/ 5% CO_2_ incubator. TNF-α (20 ng/ml) was used to stimulate human RA FLS to dig the associated RA mechanism.

**Micro-computed tomography (Micro-CT) analysis**

By the end of the treatment period, the animals were humanly dispatched, and the left hind paw was amputated and fixed in 4% paraformaldehyde (PFA), and then scanned by using Micro-CT scanner (SkyScan 1176, Bruker, Belgium). We used the following scanning parameters in order to get high-quality images of the rat’s joint: 53 kV, 470 μA, 65 ms exposure time, 0.7, rotation step in 360°, and a 1 mm Al filter. The images were reconstructed by using NRecon software (Bruker-micro CT, Belgium)

**Plasmid constructs and transfection**

STAT3 overexpression vector complementary DNA (cDNA) was constructed by BersinBio Biotech Company (Guangzhou, China) through the insertion of human STAT3 cDNA into the pcDNA3.1 vector. FLS were transfected with this cDNA constructs with Lipofectamine 3000 (Invitrogen, CA, USA) in compliance with the manufacturer’s protocol. Control group was the cells transfected with empty vectors.

**Real-time quantitative PCR (RT-qPCR) analysis**

Total RNA from pretreated human RA FLS was extracted by TRIzol Reagent (TaKaRa Biotechnology, Dalian, China). Total RNA (1.0 μg) of each sample was reverse transcribed into cDNA by BIO-RAD cDNA Synthesis Kit (CA, USA) based on the manufacturer’s protocol. The expression of target gene was measured by semi-quantitative qPCR through using iTaqTM Universal SYBR Green Supermix (BIO-RAD, CA, USA). RT-qPCR experiment was conducted by ViiA™ 7 Real-Time PCR System (CA, USA) with initial denaturation at 95° C for 10 min and then 40 cycles of denaturation at 95°C for 30 s, and then annealing at 60° C for 30 s, 72° C for 30 s. GAPDH was used as the reference gene for analyzing the relative target gene expression by using the 2^-△△Ct^ method. The target gene primers in the RT-qPCR reaction were list in Table. S2.

**Small interfering RNA (siRNA) transfection**

Human NAV2 small interfering RNA (commercial si NAV2, sc-96275), human STAT3 small interfering (commercial si STAT3, sc-29493), and control siRNA (si Scr) were produced by Santa Cruz Biotechnology, Inc. (CA, USA). In order to introduce siRNA into cells, FLS were planted on the 6-well plate at 30-50% confluence before transfection. Individual siRNA (at 25-50 nM), Lipofectamine RNAiMAX (Invitrogen, CA, USA), and Opti-MEM (Thermo Fisher Scientific, MA, USA) were mixed and incubated at room temperature for 10-15 min. siRNA Lipofectamine RNAiMAX complexes were added into cells for 48 h and then the medium was replaced by fresh serum DMEM after the transfection. Experiments were conduct after transfection for 60 h.

**Western blot analysis**

Cells and tissue proteins were lysed in RIPA buffer (Cell Signaling Biotechnology, MA, USA) which contains protease and phosphatase inhibitor cocktail (Sigma, St Louis, USA). The whole-lysate samples were isolated by SDS-PAGE and after that were transferred onto nitrocellulose membranes for immunoblot analysis. These membranes were blocked with 5% non-fat milk in Tris-buffered saline with Tween 20 (TBST) for 1 h at room temperature and after that incubated with the indicated primary antibodies overnight at 4℃. The next day, membranes were washed 3 times in TBST for 5 min on a rotating platform and then incubated with an anti-rabbit or anti-mouse secondary antibody for 1 h at room temperature. Finally, membranes were washed 3 times using TBST and all bands in the membranes were imaged by GE Amersham Imager 600 machine (IL, USA). The intensity of each band was analyzed by Image J software (MD, USA). GAPDH was regarded as a loading control.

**Immunofluorescence staining analysis**

FLS were seeded on the glass coverslips which were previously placed in 6-well plates. After different treatments, cells were fixed by using 4% paraformaldehyde for 20 min, and then permeabilization with 0.3% Triton X-100 in PBS for 10 min. Then the slides were blocked in PBS with 1% BSA for 1 h at the room temperature, and then incubated overnight with primary antibodies at 4°C. The next day, appropriate secondary antibodies were added in and incubated for 2 h at the room temperature. The nuclei were stained with DAPI for 5 min. Finally, all images were captured by using a fluorescence microscope (Olympus IX73, Tokyo, Japan).

**Proliferation assay**

FLS were seeded in 96-well plates at a density of 5.0×10^3^ cells/ well and then cultured overnight for cell adhesion. The cells were treated with siRNA negative or si NAV2 for 48 h and then induced by TNF-α for 12 h. The BrdU assay was performed to examine cell proliferation according to the manufacture’s instruction book. The absorption value was measured at 450 nm by using SpectraMax Paradigm (Molecular Device, CA, USA). The whole values were standardized by comparison with the data from the untreated cells.

**Invasion assay**

After the transfection with si NAV2 or si Scr for 48 h, FLS were subjected to invasion assay. For the *in vitro* invasion assay, Matrigel basement membrane matrix (BD Biosciences, Oxford, UK) was pre-coated on the membrane of the upper chamber and 5×10^4^ cells were re-suspended in 200 μl of the medium with 1% FBS, and then seeded in the upper chamber, while the lower chamber was filled with complete medium. Cells were seeded as a migration assay based on our previously described method.^3^ Finally, the stained cells were counted as the mean number of cells per 6 random fields for each assay. Experiments were replicated for 3 times.

**Wound Healing Assay**

Human RA FLS were planted into a 6-well culture plate and grown to confluence up to about 60%. After that the cells were serum-deprived and treated with NAV2-siRNA or si Scr. Then the medium was replaced with fresh DMEM within TNF-α (20 ng/ml) stimulation. The plate was scratched with a sterile plastic pipette tip and washed with PBS 3 times to remove the deciduous cells. At the present, a single wound was created in the center of the cell monolayer. After 12 h TNF-α stimulation, the wound areas were respectively photographed by using a microscope (Olympus IX73, Tokyo, Japan) equipped with a digital camera. Three assays of wound area were made at random fields. The extent of wound closure was presented as the percentage by which the original scratch area had decreased at each measured time point. All the data are obtained from three independent experiments.

**EdU analysis on human RA FLS**

To investigate cell proliferation, an immunofluorescence assay was performed to detect 5-Ethynyl-2’-deoxyuridine (EdU) incorporated into cellular DNA (with Flur488 Click-iT EdU Kit, KeyGEN BioTECH, Jiangsu, China). The immunofluorescence examination was conduct according to the manufacturer’s instruction book. All the cellular nuclei were stained with DAPI. Olympus inverted fluorescent microscope was used to detect immunofluorescence EdU positive cells.

**Luciferase reporter assay**

The fragment containing the core promoter region of NAV2 (-2000 - +500) was inserted between the XhoI and HindІІІ sites of the firefly luciferase vector pGL4.10 (Promega, WI, USA). The NAV2 promoter-luciferase reporter plasmids were constructed by BersinBio (Guangzhou, China). 293T cells were transfected with either of these plasmids (together with a plasmid containing the Renilla luciferase gene to serve as a reference for transfection efficiency), with or without co-transfection of the plasmid to overexpress STAT3. After 48 h transfection and then induction with TNF-α for 12 h, the firefly luciferase activity and Renilla luciferase activity were measured on a luminometer (Berthold Technologies, Germany).

**Chromatin immunoprecipitation (ChIP) assay**

ChIP assay was performed by using ChIP Kit (Bes5001, BersinBio, Guangzhou, China) according to the manufacturer’s instructions. Briefly, the cells were fixed with 1% formaldehyde, and then cross-linking was quenched by adding in 100 μl of 1.375 M glycine per milliliter of culture. The samples were sonicated on ice to shear the DNA into 200 to 600 bp fragments. For each total cell lysate, one third was used as the DNA input control, another third was immunoprecipitated with anti-STAT3 antibody, and the last third was subjected to non-immune rabbit IgG (Cell Signaling Biotechnology, MA, USA). The DNA fragments were purified by spin columns (Qiagen, Hilden, Germany), and then RT-qPCR assay was used to amplify the segment in the promoter region of NAV2 with the following two primers:

**Prime 1**, Forward: AATGTCCACGGTAGTGCCTC

Reverse: TGTGCTCCCCATGTCCCTTA

**Prime 2**, Forward: AGCTCCATCCAGAGAGGTGT

Reverse: CAATCATGCCTGCTAACGCC

**ChIP-seq analysis**

﻿Human primary RA FLS and human primary OA FLS were cultured until 3×10^7^ for H3K27ac ChIP-seq to identy SEs. Appropriate amount of cross-linking buffer was added into the petri dish, after that formaldehyde with a final concentration of 1% was cross-linked at the room temperature for 10 min, and then quenched with 0.125 M glycine for 5 min. Next Pour away the medium, wash the cells with the pre-cooled PBS. Add 4 ml pre-cooled PBS, then scrape the cells off with cell scraping. Cells were lysed in 1 ml lysis buffer and rotated for 30 min at 4℃. Then cell lysates were centrifuged at 2,400 g for 10 min at 4℃ to isolate the cell nuclei. After that, nuclei were suspended in the digestion buffer and subjected for enzymatic shearing to shear chromatin fragments with an average size between 200 bp and 500 bp through incubating the tube at 37℃ for 15 min. Then stop the shearing reaction, and the fragmented chromatin was centrifuged at 18000 g for 10 min at 4℃. The supernatant was transferred to a new tube and added the ChIP reaction mix containing protein A/G magnetic beads, ChIP IP buffer, antibody, protease inhibitor cocktail, and then rotated at 4℃ overnight. After overnight incubation, washed protein A/G magnetic beads. The chromatin was eluted in the reverse cross-linking buffer followed by incubating the tube at 65℃ for 3h. Then the ChIP DNA was treated with RNase A and proteinase K at 37℃ for 30 min and purified by using Phenol chloroform. Finally, ChIP DNA was processed for library generation by using the QIA seq Ultralow Input Library Kit (QIAGEN) based on the manufacturer’s instruction book.

In order to define typical and super enhancers, we used the ROSE software. Firstly, the region of enhancer was identified. Then the enrichment degree of each enhancer was calculated by the mathematical statistics. The point of slope 1 was sorted based on the enrichment of all the enhancers, as the threshold for typical enhancers and super enhancers.

**Molecular docking**

The two protein crystal structures of NAV2 and STAT3 (PDB ID: 2YRN, 6NJS) were downloaded from the protein database (https://www.rcsb.org). The two protein structures were optimized on the molecular operating environment platform (MOE 2019.1). Then set the two proteins contact sites to the full surface and set the conformation after docking. We used the London dG scoring function to select the conformation with the highest level of the negative energy.

**Molecular Dynamics Simulation**

It is vital to figure out the stability of the interaction of NAV2 and STAT3 in order to further evaluate the binding affinity. According to the molecular docking results, the best binding pose of NAV2 and STAT3 was subjected to MD simulation by using GROMACS package version 2020.3.^4^ The selected binding pose of NAV2 and STAT3 protein were converted to Mol file format by using Avogadro software. The topology of the proteins was generated with the General Amber Force Field (GAFF) ^5^ to assign atom types and bonded parameters. Before the simulation, the systems were first equilibrated through 0.1 ns in NVT, NPT ensemble at 300 K and 1 atm, then applying the position restraints on the proteins with periodic boundary conditions. Then the MD simulation was executed to 40 ns time. Root mean-squared deviation (RMSD) value was stored in the trajectory for every 2 fs and analyzed data through using Grace Software.^6^

**Statistical analysis**

The statistical analysis was performed by using the GraphPad Prism version 8.0. All the values are expressed as the mean ± SEM or mean ± SD. The two-sample *t*-test for comparing the means of two groups, and the one-way ANOVA with Dunnett’s multiple comparisons for comparing means among multiple groups. *P* < 0.05 was considered as statistically significant.
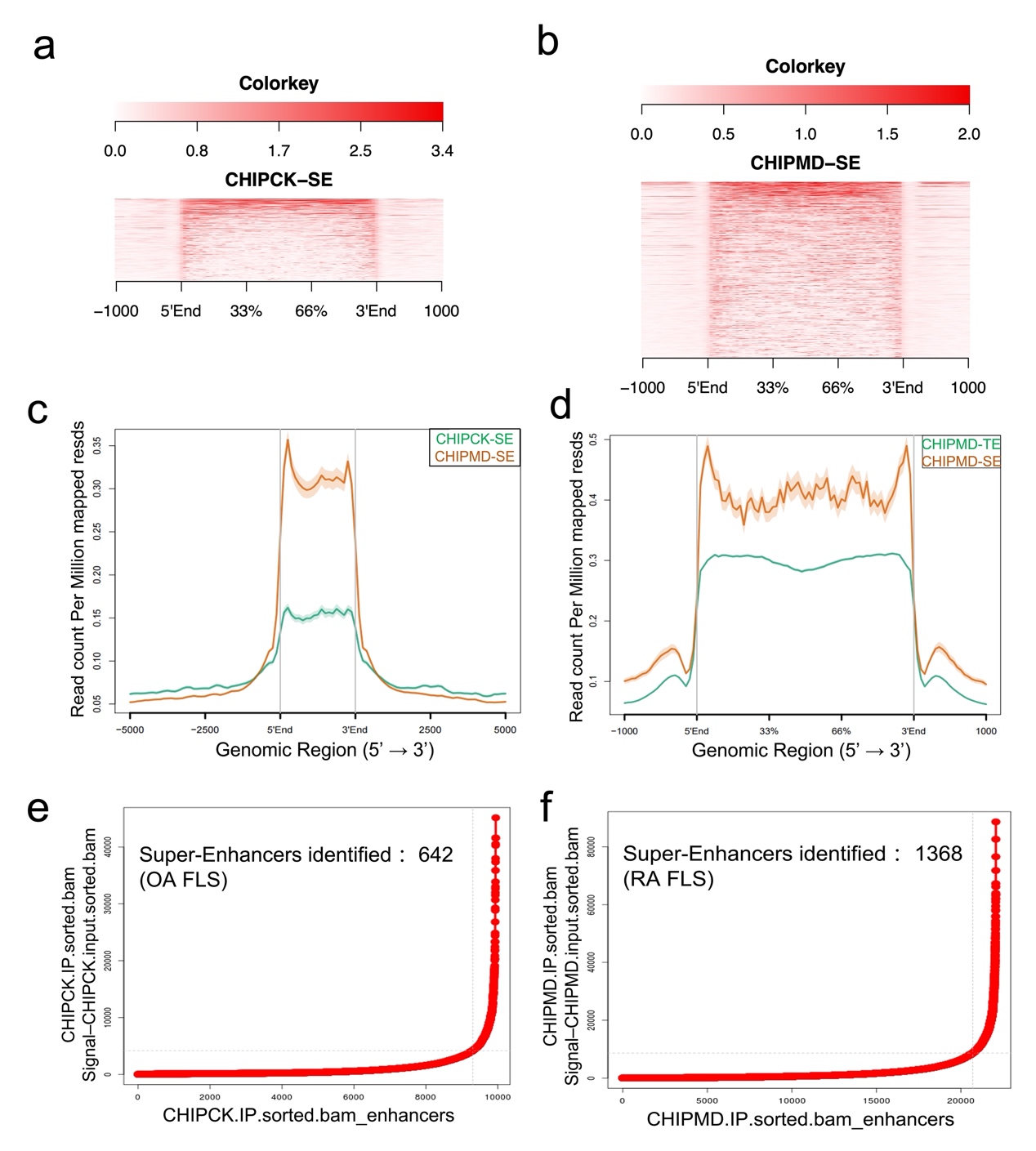


**Figure. S1. The identification of SE in RA FLS and OA FLS. a, b** Increased ChIP-seq signal of SE in human RA FLS (CHIPMD) compared to human OA FLS (CHIPCK). **c** The signal strength of SE was detected in human RA FLS and OA FLS. **d** The enrichment level of typical enhancers and SEs in the coding gene region in human RA FLS. **e, f** SEs were identified in OA FLS and in RA FLS.


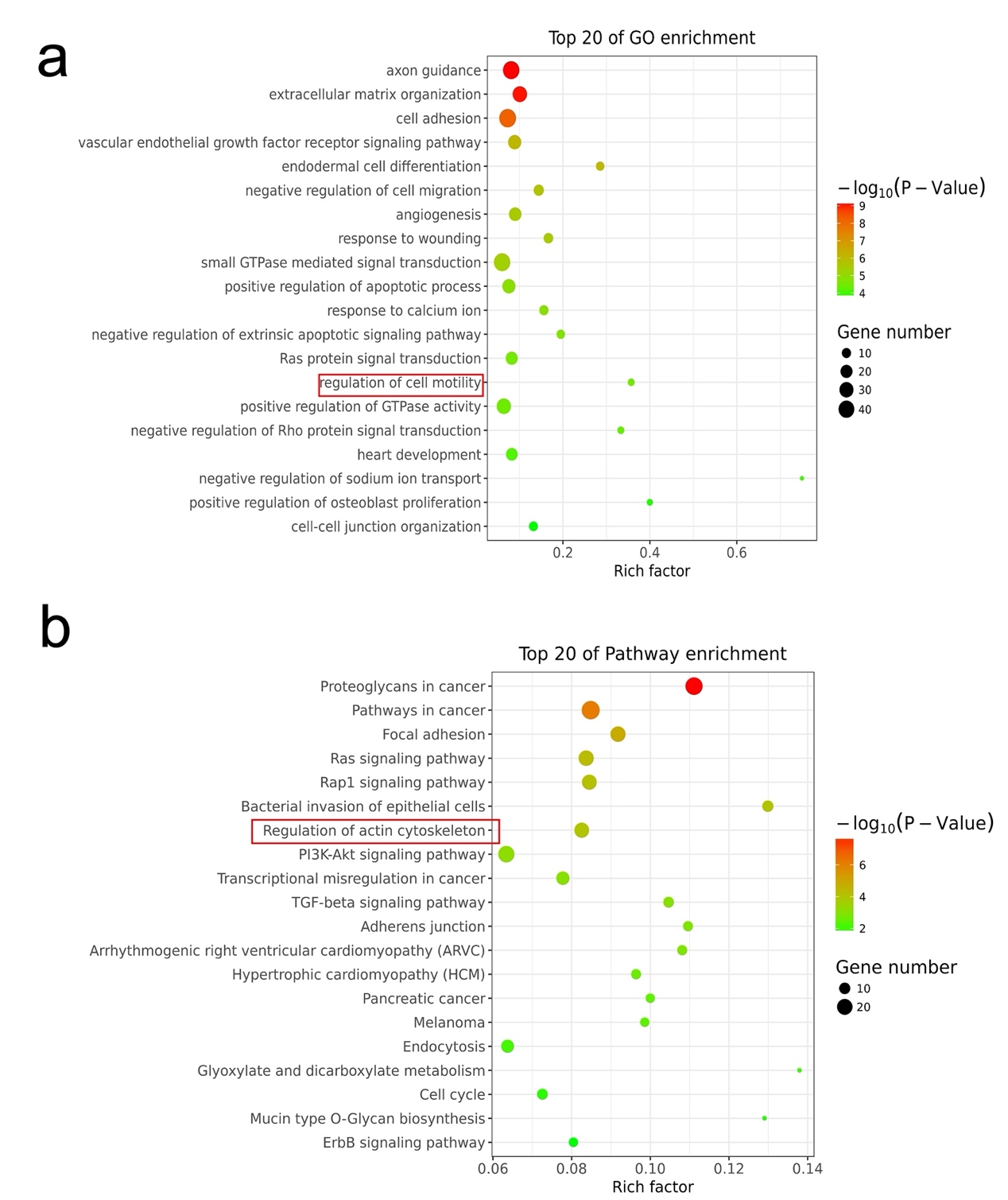


**Figure. S2. GO analysis and pathway enrichment in RA.** **a** GO analysis of genes with differential enrichment of SE. **b** Pathway analysis with differential enrichment of SE.


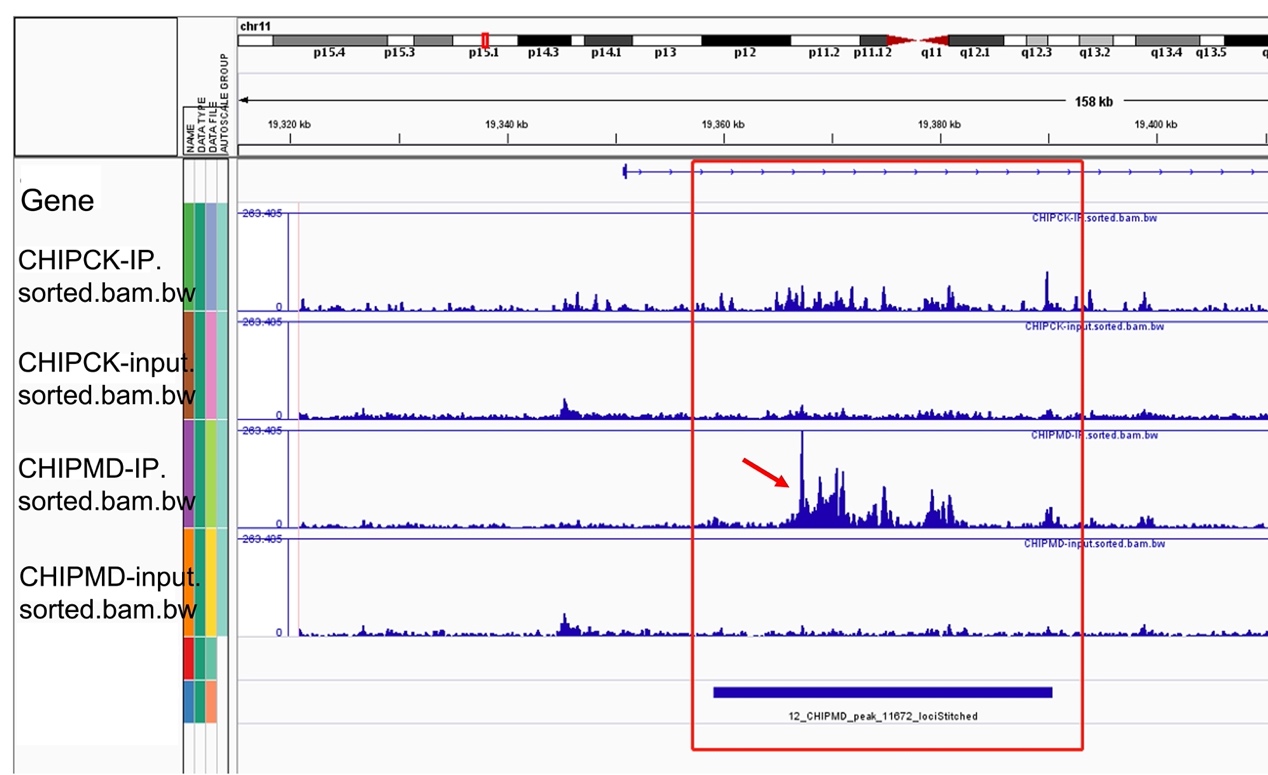


**Figure. S3. SE signal of NAV2 showed a significant increase in primary human RA FLS.**

**
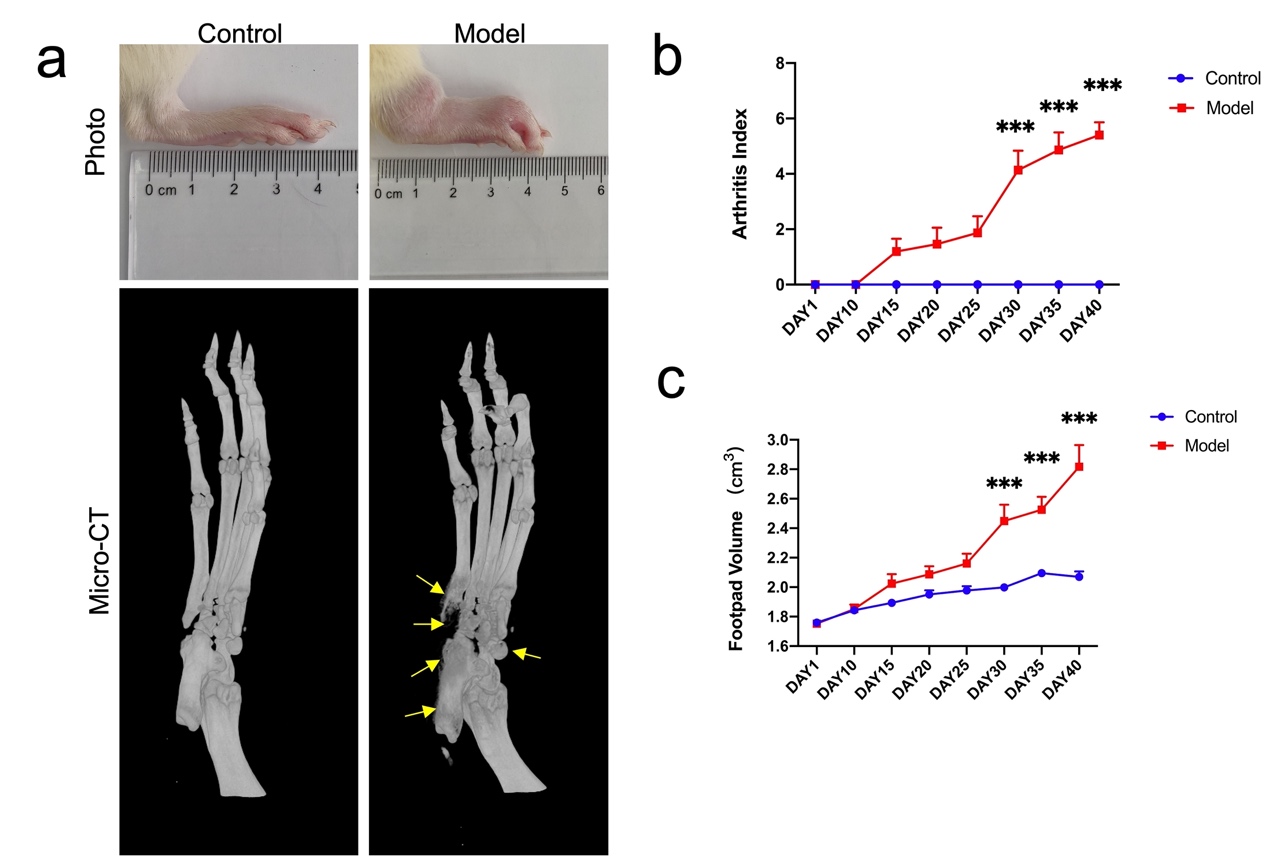
**

**Figure. S4. Establishing AIA rats’ model. a** Photos and Micro-CT images of ankles from the Control and AIA rats at day 30 after immunization were shown. Arthritis index (**b**) and increased hind paw volume (**c**) were shown (n = 15 for each group). *** *P* *<* 0.001.


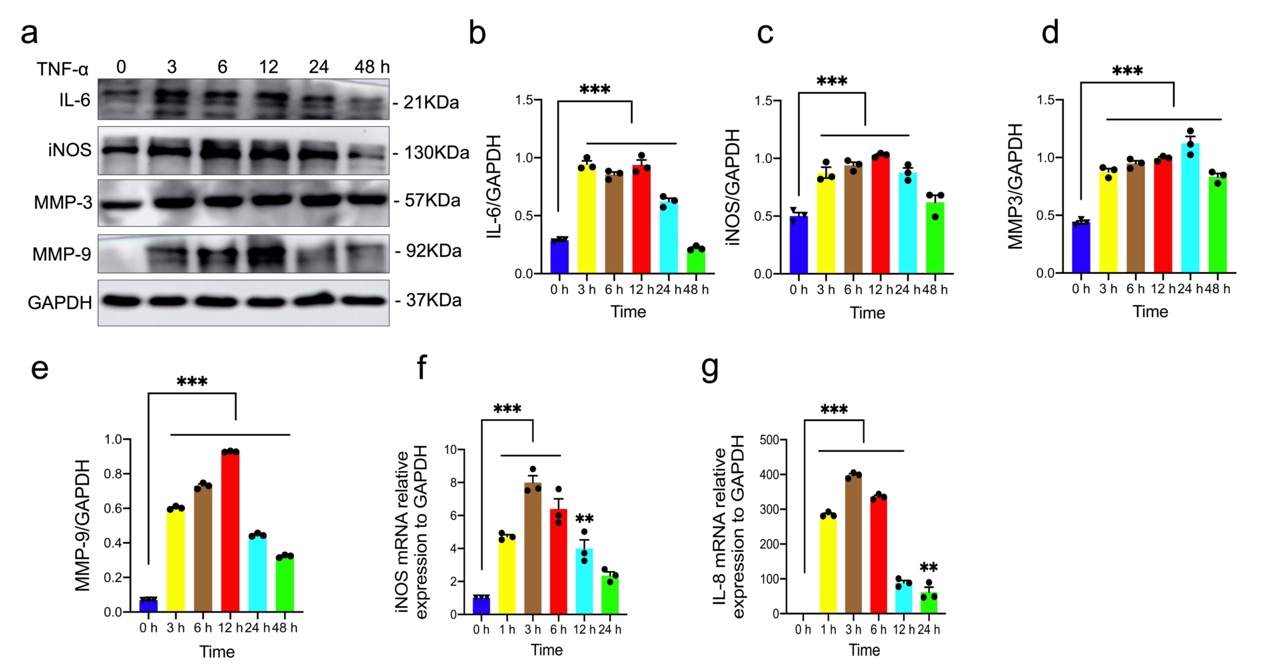


**Figure. S5. ﻿Establishing an inflammatory response in TNF-α-induced FLS.** Human RA FLS were treated with TNF-α (20 ng/ml) for 0, 1, 3, 6, 12, 24 and 48 h, the inflammatory mediator levels were analyzed as described in Materials and methods, respectively. **a-e** IL-6, iNOS, MMP-3 and MMP-9 protein expression, GAPDH was used as loading control. **f, g** iNOS, IL-8 mRNA expression. All the data from 3 independent experiments were presented as means±SEM, *** *P* *<* 0.001.


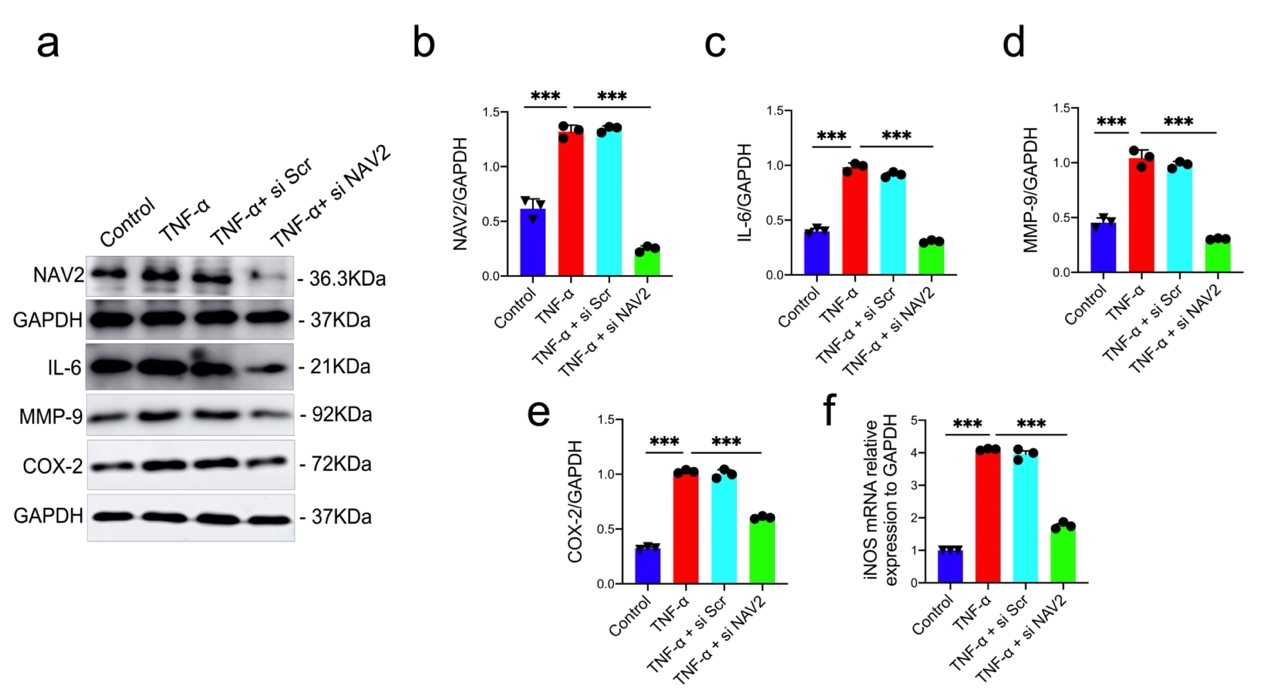


**Figure. S6. NAV2 positively regulates the inflammatory response in RA.** FLS were transfected with si Scr or si NAV2 prior to treatment with TNF-α (20 ng/ml), the level of protein expression was determined by Western blotting after 12 h stimulation. **a-e** NAV2 silencing resulted in decreased protein expression of IL-6, MMP-9, and COX-2. **f** the mRNA expression level of iNOS were measured by RT-qPCR after stimulation for 3 h. Data are presented as means ± SEM from at least 3 independent experiments. *** *P* < 0.001.


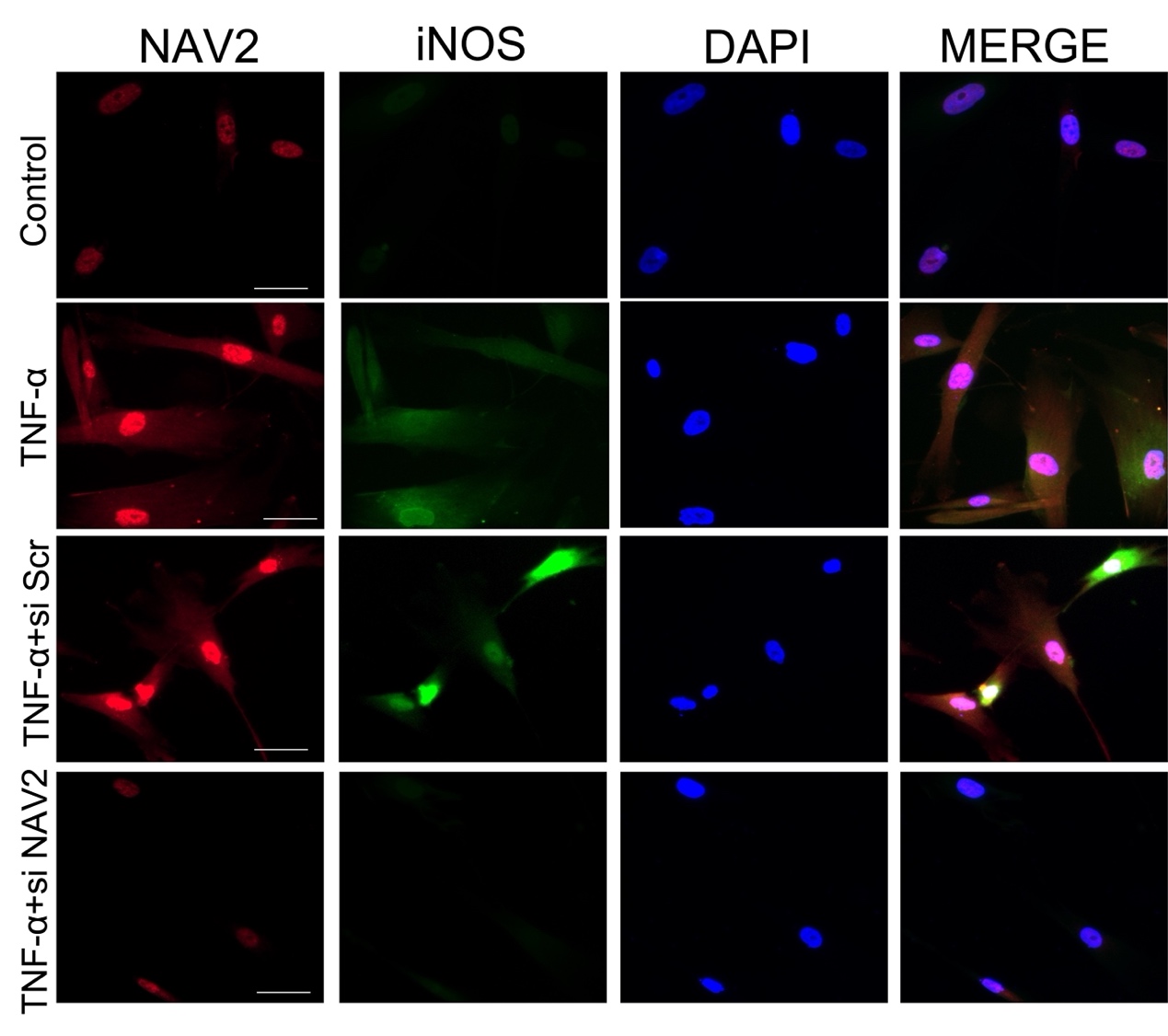


**Figure. S7. Cells were subjected to immunofluorescence staining for NAV2 and iNOS.** DAPI for stain nuclei (blue). NAV2 and iNOS were stained by red and green, respectively. Scale bars, 50 μm.


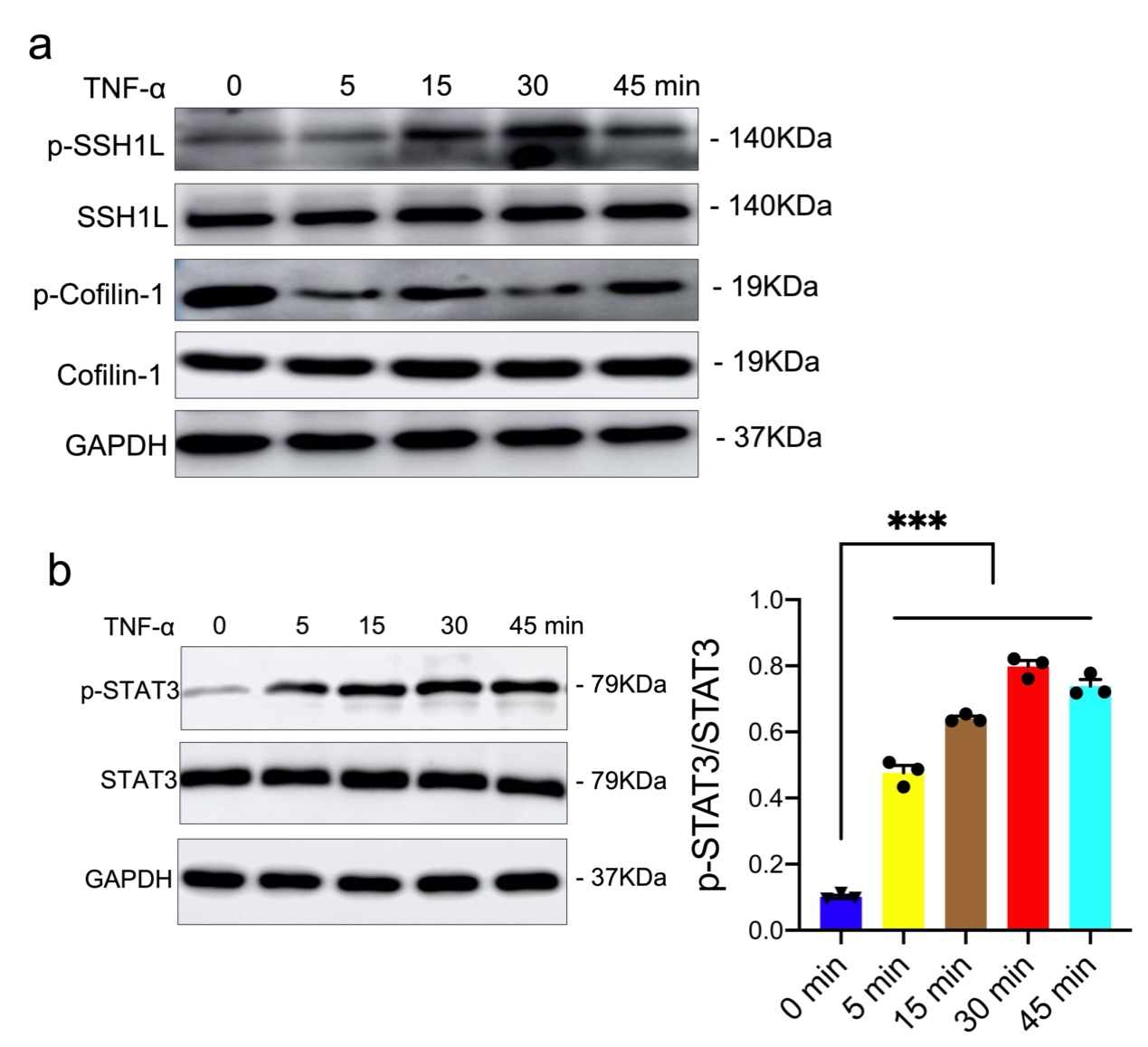


**Figure. S8. SSH1L/Cofilin-1 pathway and transcription factor STAT3 could be activated by TNF-α in RA FLS. a** RA FLS were stimulated with or without TNF-α (20 ng/ml) for the indicated periods, the SSH1L/Cofilin-1 pathway was analyzed as described in Materials and Methods, respectively. Protein levels of p-SSH1L, SSH1L, p-Cofilin-1 and Cofilin-1 in FLS treated with TNF-α by using Western blot. **b** Increased expression of p-STAT3 at protein level in TNF-α-induced FLS. GAPDH serves as the loading control, blots shown are representative of data from at least three different replicates. *** *P* < 0.001 vs unstimulated cells.


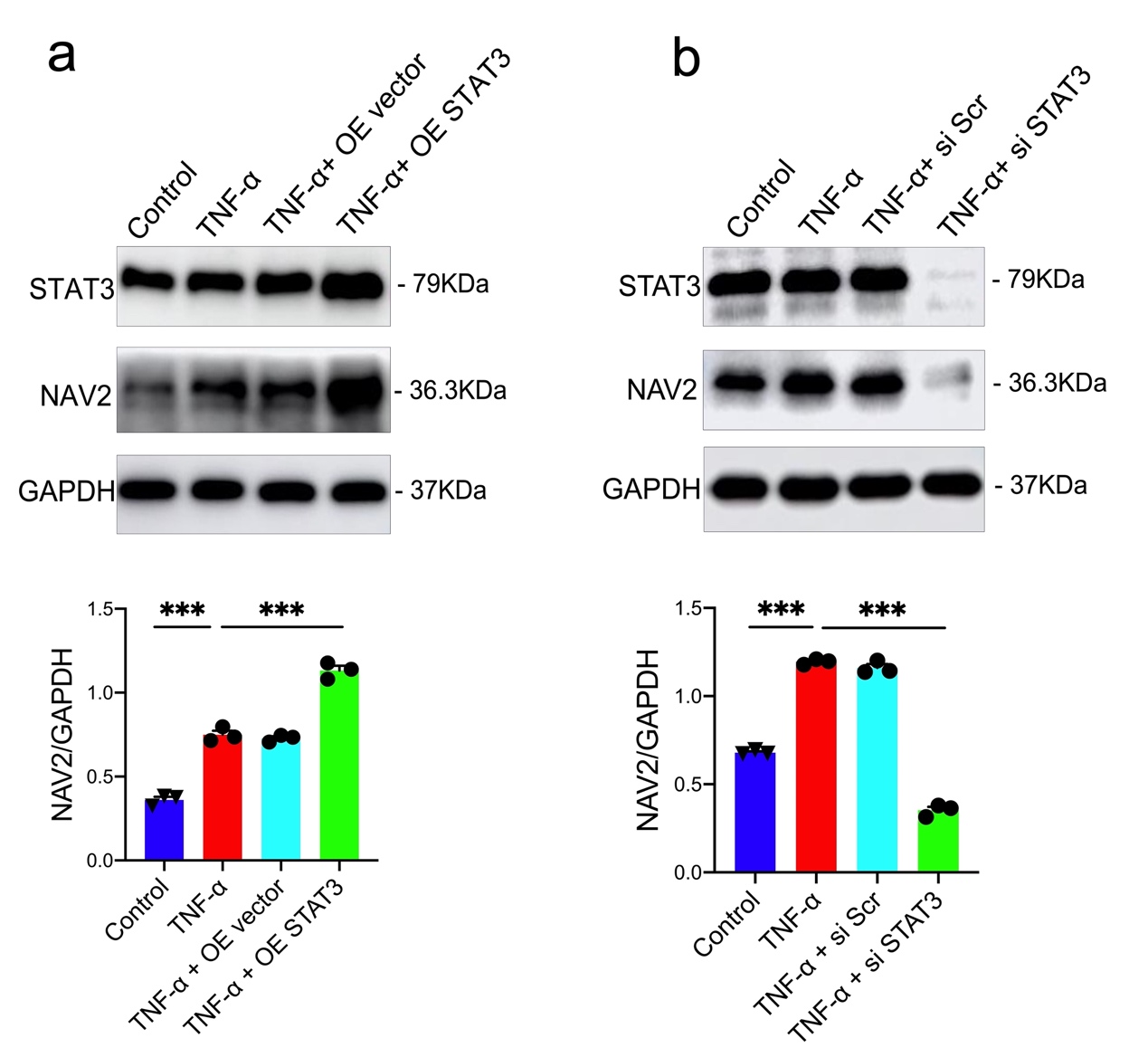


**Figure. S9. NAV2 expression could be regulated by STAT3. a** The expression of STAT3 was validated in human RA FLS transfected with STAT3-cDNA (OE STAT3) or control empty vector (OE vector). Overexpression (OE) of STAT3 was confirmed by Western blot and NAV2 level increased in STAT3 overexpressing cells, all blots shown are representative images from at least three replicates. **b** STAT3 knockdown (si STAT3) reversed the NAV2 expression level in TNF-α-induced FLS. GAPDH serves as the loading control, blots shown are representative of data from at least three different replicates. *** *P* < 0.001.


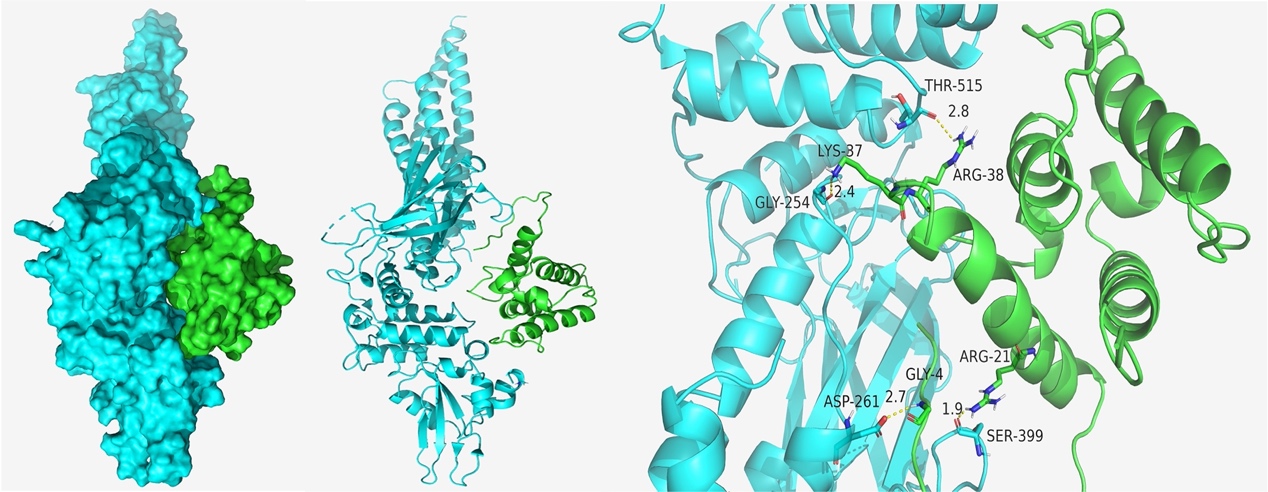


**Figure. S10. Molecular docking analysis of STAT3 and NAV2.**


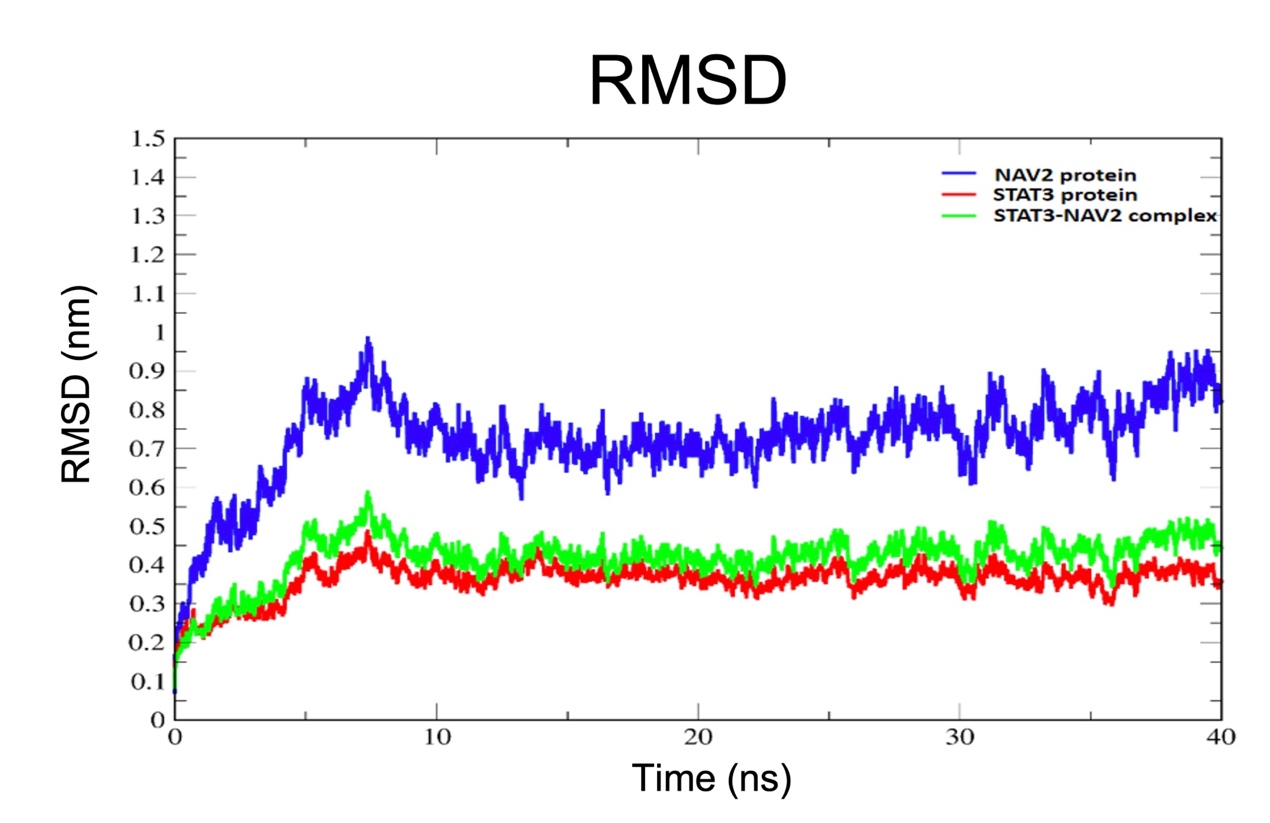
**Figure. S11.** **RMSD analysis of STAT3 and NAV2.**

**Table S1. The docking result of two target proteins**

| Protein 1 | Protein 2 | Binding Energy (kcal/mol) |
| --- | --- | --- |
| NAV2 | STAT3 | -65.05 |

| Gene name | Primer name | Primer sequence (5’ to 3’) |
| --- | --- | --- |
| NAV2 | Homo _NAV2_F | GAGGGACGGGAGTTGACAGA |
|  | Homo _NAV2_R | CAGTTGAGCAGCCCATTGAA |
| GAPDH | Homo _GAPDH_F | GATTCCACCCATGGCAAATTCC |
|  | Homo _GAPDH_R | GCATCGCCCCACTTGATTTT |
| IL-8 | Homo _IL-8_ F | ACACTGCGCCAACACAGAAA |
|  | Homo _IL-8_ R | GTTTTCCTTGGGGTCCAGACA |
| iNOS | Homo _GAPDH_ F | TTCAGTATCACAACCTCAGCAAG |
|  | Homo _GAPDH_ R | TGGACCTGCAAGTTAAAATCCC |

**Table S2. Primers used for qRT-PCR validation**

**REFERENCES**

1. Wu, W. et al. S-propargyl-cysteine attenuates inflammatory response in rheumatoid arthritis by modulating the Nrf2-ARE signaling pathway. *Redox Biol* **10**, 157-167 (2016).

2. Tan, F. et al*.* Neuron navigator 2 overexpression indicates poor prognosis of colorectal cancer and promotes invasion through the SSH1L/cofilin-1 pathway. *J Exp Clin Cancer Res* **34**, 117 (2015).

3. Wu, W. et al. Cystathionine-gamma-lyase ameliorates the histone demethylase JMJD3-mediated autoimmune response in rheumatoid arthritis. *Cell Mol Immunol* **16**, 694-705 (2019).

4. Messias, A. et al. Out of Sight, Out of Mind: The Effect of the Equilibration Protocol on the Structural Ensembles of Charged Glycolipid Bilayers. *Molecules* **25**(2020).

5. Loschwitz, J. et al. Dataset of AMBER force field parameters of drugs, natural products and steroids for simulations using GROMACS. *Data Brief* **35**, 106948 (2021).

6. Sinha, S. & Wang, S. Classification of VUS and unclassified variants in BRCA1 BRCT repeats by molecular dynamics simulation. *Comput Struct Biotechnol J* **18**, 723-736 (2020).
